# A Positive or Negative Connection of Diabetes Mellitus to the Oral Microbiota

**DOI:** 10.5152/eurasianjmed.2023.21164

**Published:** 2023-02-01

**Authors:** Ali Abdul Hussein S. AL-Janabi

**Affiliations:** 1 Department of Microbiology, University of Karbala Faculty of Medicine, Karbala, Iraq

**Keywords:** Diabetes mellitus, oral, microbiota, periodontal disease, candidiasis

## Abstract

Common noncommunicable diabetes mellitus disease has many complications in several parts of the human body. The oral cavity is one of the areas affected by diabetes mellitus conditions. The most common complications of diabetes mellitus in oral areas include increased drying of the mouth and increased oral diseases resulting from either microbial activity, such as dental caries, periodontal diseases, and oral candidiasis, or physiological problems, such as oral cancer, burning mouth syndrome, and temporomandibular disorders. Diabetes mellitus also has an impact on the diversity and quantity of oral microbiota. Oral infections promoted by diabetes mellitus mainly result from disturbance of the balance between different species of oral microbiota. Some oral species may be positively or negatively correlated with diabetes mellitus, while others may not be affected at all. The most numerous species in the presence of diabetes mellitus are those of phylum Firmicutes of bacteria such as hemolytic *Streptococci*, *Staphylococcus* spp., *Prevotella *spp.*, Leptotrichia* spp., and *Veillonella* and species of the fungus *Candida*.* Proteobacteria* spp. and *Bifidobacteria* spp. are common microbiota that are negatively impacted by diabetes mellitus. In general, the effect of diabetes mellitus could include all types of oral microbiota, whether it is bacteria or fungi. The 3 types of association between diabetes mellitus and oral microbiota that will be illustrated in this review are increase, decrease, or lack of impact. As final inclusion, a great number of oral microbiota have increased in the presence of diabetes mellitus.

**Main Points**

- Correlation between diabetes mellitus (DM) and oral microbiota can take 3 ways: positive, negative, and ineffective.
- The main role of DM is to convert oral commensal microbiota into pathogens.
- The diversity and number of oral microbiota increase with DM.
- Diabetes mellitus is effective on all types of oral microbiota with no difference.

## Introduction

Diabetes mellitus (DM) which is characterized by high levels of sugar in the blood due to an alteration in the metabolism of carbohydrates, fats, or proteins is widely distributed worldwide.^1,2^ The prevalence and incidence of DM are growing at a rapid rate each year. In 1985, DM counted 30 million persons, which increased in 2000 to 150 million and in 2007 to 246 million.^3^ Diabetes mellitus can take many forms depending on the causal default. Type 1 DM (T1DM) results from a failure to secrete insulin and type 2 DM (T2DM) is most commonly represented by insulin-resistant cells.^4,5^

The oral cavity contains a great number of microorganisms of high diversity.^6,7^ This makes it the second largest reservoir of different types of microbial communities.^6,8^ Several favorable oral conditions may support the life of the microbiota such as the structural nature of the oral parts and internal oral conditions.^9,10^ The presence of microbiota in the oral cavity provides numerous advantages for the human body such as protection against infection by pathogenic microorganisms through competition, supplying humans with vitamin B12, and digestion of some type of nutrients.^6,7,10^

Diabetes mellitus has numerous complications on the oral cavity represented by the increased pathogenic activity of the oral microbiota to cause diseases such as dental caries, periodontal diseases (PD), and oral candidiasis or cause physiological disorders such as dry mouth (xerostomia and decreased salivary flow rate), lesions in oral mucosa, altered taste, geographic tongue, oral cancer, burning mouth syndrome, and temporomandibular disorders.^11-13^

Three actions may be performed by DM on the oral microbiota: increase, decrease, or not have any effect on the growth of the oral microbiota. In general, diabetes frequently increases the number of oral microbiota which leads to its diversification.^14-16^ Common bacterial species found in patients with DM compared to patients without DM or healthy people include hemolytic Streptococci, *Staphylococcus* spp., *Prevotella *spp*.,* *Leptotrichia* spp., and *Veillonella* spp.^17^ Other species may not be different in either DM patients or non-diabetic patients such as *Prevotella copri*, *Alloprevotella rava*, and *Ralstonia pickettii*.^18^ Meanwhile, the *Proteobacteria *species is not found in the patient with DM, whereas it is clearly observed in healthy people.^18,19^

Diabetes mellitus may also encourage different fungal species like *Candida* to become pathogenic due to many DM-related factors such as xerostomia, low salivary flow, higher salivary glucose level, and microvascular degeneration.^2,20^ *Candida albicans* is the most frequent species of *Candida* in diabetic patients compared to non-diabetic individuals.^21-24^ The correlation of DM with oral microbiota is discussed in this review.

### Diabetes Mellitus

Diabetes mellitus is a common physiological disorder characterized by chronic hyperglycemia resulting from partial or complete impairment in the metabolism of carbohydrates, proteins, or lipids.^1,2^ Thus, DM is not a single disease, but it includes all disorders leading to increased blood sugar levels.^13,25^ It is found throughout the world, whether developed or developing.^4^ The prevalence of DM in developed countries is higher than in developing countries and higher among females than males.^26^ The incidence of DM always goes up over time. It was estimated to be 30 million in 1985 and increased to 150 million in 2000 and 246 million in 2007 and is expected to reach 380 million in 2025 as predicted by the International Diabetes Federation.^3^ The figure is also expected to rise from 135 million in 1995 to 300 million in 2015, particularly in developing countries.^26^ In the Eastern Mediterranean region, DM constituted the fourth cause of death, particularly type 2.^27^ The epidemic of DM as one of the noncommunicable diseases is a public health challenge faced by the world at present.^27^

The main reasons for DM are the default in insulin secretion, action, or both that all lead to disruption in glycemic control.^4,13,25,28^ Thus, many types of DM can be recognized, including T1DM which is common in children and results from a defect in insulin secretion due to the destruction of insulin-producing pancreatic beta-cells, type 2 (T2DM) which is common in adults and results from insulin resistance, gestational type (high glucose level during pregnancy), and other types that may result from the effect of genetic defects, environment, infections, or from drugs effects.^5,26,29,30^ Type 2 DM occurs more frequently than other types, accounting for 90% of all DM cases.^26^ Initiation of T2DM is primarily related to physiological changes in mitochondrial metabolism, such as the production of adenosine triphosphate, high oxidative indicators, and changes in mitochondrial RNA level.^2^

Several factors are associated with the development of DM and the determination of its type, such as genetic predisposition or environmental factors that induced an autoimmune destruction of beta cells to form T1DM or insulin-resistant cells as with T2DM.^4,5^ Type 1 DM is mostly induced by multifactorial events ranging from autoimmune disease which is mediated by antibodies or exposure to toxins in the environment to genetic predisposing factor represented by the presence of HLA-DR/DQ polymorphism of major histocompatibility complex (MHC) class II histocompatibility complex.^2^ The evolution of DM is more related to age; T2DM is more frequent in adults, whereas T1DM is frequent in children.^1^ In addition, prevalence of DM is estimated to be 11.3% of all people aged 20 and older and about 26.9% of people aged 65 and older.^13^ In all men, about 13 million or 11.8% of them aged 20 years or older suffer from DM.^13^ Other factors may include obesity, diet, unhealthy lifestyle, physical inactivity, hormonal activity, drugs, and infections.^3-4,27,29^

### Oral Normal Flora

Microorganisms have a long history of relationship with humans over millions of years. They establish a symbiosis coexisting with various parts of the human body as a microbiota.^6,7^ The mouth is considered the second largest reservoir of different types of microbial communities, including bacteria, fungi, and viruses.^6,8^ It contains over 700 species of microorganisms with more than 500 bacterial taxa and about 22 identified predominant genera colonizing parts of the oral cavity.^6-8,10^ The first source of oral microbiota in the human body comes from contact with the birth canal at birth, followed by parents, and later from other sources such as water, food, and the surrounding environment.^8-10^ The density and diversity of microorganisms increase over time in the oral cavity, moving from optional in childhood to very high in adults.^10^

The oral cavity has many favorable conditions to support the life of different types of microorganisms like the structural nature of oral parts and suitability in conditions for microbial retention and growth.^9-10^ The soft tissues of the mucosa and hard surfaces of the teeth, tongue, gingiva, and palate provide suitable sites for colonizing and stabilizing the oral microbiota.^6^ A diet rich in carbohydrates that degrade to simple sugar and saliva compositions of mineral, ionic, and organic compounds with its buffering capacity are all factors that regulate the presence of oral microbiota.^10^

The oral microbiota is a complex community that is concerned with being stable in diversity and density with a chance for some species to experience minor changes in location and time.^7,10^ The oral microbiota can be divided into 2 types according to the time of appearance as resident and transient or divided according to the source of entry as indigenous and exogenous or supplemental.^7^ The indigenous oral microbiota is most often represented in large numbers (>1%), in particular at the location of the supragingival plaque and the surface of the tongue, while the transitional group is usually in smaller numbers (<1%).^7^ However, oral conditions are the effective factor in determining the type of microorganism in the site of the oral cavity that may later be associated with the change in oral state.^9^

The presence of microflora in the oral cavity brings many benefits for the human body. The principal role of the oral microbiota is to provide natural protection to the oral cavity against the pathogenicity of many pathogenic organisms.^6,10^ This type of protection typically emerges from competing with other organisms. It envisions an oral microbiota protection key to control or destroy other enclosed microorganisms and immunization against numerous diseases potentially caused by oral pathogens.^10^ Oral microbiota may gain competition by producing various metabolites such as volatile fatty acids and hydrogen or by modifying the conditions of the local oral environment such as pH and redox potential for suppressing other microorganisms.^10^ Thus, the presence of microbiota plays an important role in maintaining human health because it is associated with the first line of defense against pathogens that cause disease in either oral cavity such as dental caries and PD or in other internal parts of the human body such as colorectal cancer, pancreatic cancer, and inflammatory bowel syndrome.^8,10^ However, any disturbance or alteration of the oral microbiota community which is called dysbiosis will affect the balance rate with other closed microorganisms and give the pathogen group an opportunity to exceed in number and cause various diseases.^9^ In general, infection in the oral cavity usually starts with the adhesion of the pathogens to the surface of the mucosa to prevent their removal by the mechanical action of saliva and by the defense of the host, thus allowing them to penetrate deep into the oral tissue.^10^

Other benefits of oral microbiota to the human body include providing vitamin B12 to the host as a product from many lactic acid bacteria and *Bifidobacterium* and also facilitating digestion of some nutrient types that could not be digested by the host.^9^

### Diabetes Mellitus and Oral Infectious Diseases

Diabetes mellitus has an impact on various organs of the human body, without regional or partial exceptions. A number of complications with long-term effects are most often associated with permanent diabetes, such as neuropathy, nephropathy, retinopathy, and vascular system change.^2^ These complications result primarily from numerous destructive activities induced by DM, including inflammation, ischemia, and increased reactive oxygen species (ROS).^11^ The oral cavity containing highly vascularized and innervated sites makes it more susceptible to DM effects.^11^ Thus, many oral complications of DM can be noticed in the oral cavity, including dental caries, PD, oral candidiasis, dry mouth (xerostomia and decreased salivary flow rate), lesions in oral mucosa, altered taste, geographic tongue, oral cancer, burning mouth syndrome, and temporomandibular disorders.^11-13^ Also, oral complications of DM play a significant role in increasing the severity of DM by increasing blood sugar levels.^12^

The proposed mechanisms for the role of DM in the reduction of saliva (xerostomia) are related to the neuropathic complication of DM through a decrease in the regulation function of the autonomic nervous system on saliva production from the parotid gland.^11^ There is a decrease in saliva flow and foam in children with DM compared to the healthy group.^31^ This decrease in salivary flow or its amount will reduce the antimicrobial effects of saliva and increase the rate of infections.^12^

Strong evidence was provided to support the positive effects of DM on the initiation or progression of different bacterial and fungal infections.^12^ Dental caries, PD, and oral candidiasis are the most well-known oral infectious diseases caused by DM disorders.^2,11,12,32^ Hyperglycemia in the DM patient induces an increase in saliva glucose levels that becomes a good nutrient for cariogenic bacteria in the dental biofilm.^11^ Comparison between saliva content in children with DM and the control group showed that saliva in patients with DM had higher levels of glucose, urea, and total protein with low calcium levels and acidic pH.^31^

Dental caries or caries is a very common type of dental disease with a high prevalence worldwide. It is triggered primarily by the process of carbohydrate fermentation by acid-generating bacteria in the oral cavity.^11^ Lactic acid produced after digestion of dietary carbohydrates by bacterial biofilm is a destructive factor of dental enamel resulting in dental caries.^2^ Although many studies indicate an increased prevalence of dental caries in DM patients, it may be difficult to explain the association between these diseases and also to determine whether the DM has a direct effect on caries development or by other factors.^11^ The effect of DM types, such as T1DM in young people or T2DM in adults, on cavities should also be determined. Among Iraqis in the city of Erbil, dental caries and gingivitis were significantly higher in T1DM patients compared to the healthy control group.^33^ Thai diabetic patients also showed a high incidence of dental caries compared to non-diabetics.^34^

Periodontal disease is one of the chronic oral inflammatory diseases characterized by the accumulation of plaque and calculus on teeth formed by anaerobic Gram-negative bacteria causing destructive inflammation in the teeth-supporting tissues and bone loss.^2,11,26,32^ It can be divided into 2 types of inflammatory diseases: gingivitis as inflammatory in the gingiva (gum) and periodontitis as destructive inflammatory in the structures surrounding the teeth such as root cementum, alveolar bone, and periodontal ligament, and it can also involve gingivitis.^11^

Periodontal disease has bidirectional relationship with DM as evidenced by many studies that have reported that these 2 diseases have a strong association in high prevalence and incidence of DM in most cases and the treatment of one can decrease another.^2,11,13,16,26^ Findings from 53 observational studies show that the prevalence of T2DM is higher in periodontitis patients, with an increased risk of 53% and vice versa when diabetes conditions increase the risk of PD by 34%.^35^ The development of PD could be expected in every patient with DM who make PD the best known complication of DM or it could be the first.^13,25,32,36,37^ It is also considered as the most common type of dental disease strongly associated with DM than gingivitis or dental caries.^38^ Diabetes induces PD through the activation of periodontal microbiota to stimulate inflammatory reactions.^25^ It is therefore expected that periodontal destruction will increase with DM.^16,17,37^ Both T1DM and T2DM have an impact on the development of PD with serious effects compared to non-diabetic peoples.^13^ In general, the severity of PD is frequently higher in patients with T2DM.^39,40^ Based on the surveillance of 14 studies, 12 studies showed that patients with T2DM had significantly lower periodontal health status, while only 1 study showed no significant correlation.^26^ This is also indicated by the results of 53 observational studies in which T2DM patients exhibited poor periodontitis.^35^ The prevalence of PD did not reveal any significant difference in patients with T1DM and healthy people when diabetes is strictly metabolically and clinically controlled,^41^ while the complication of PD has increased in patients with DM when they have uncontrolled diabetes, especially when they are older and have a longer duration of diabetes.^42^

Diabetes can have a variable effect on the development, progression, and severity of PD, which influences the correlation between these diseases.^32^ Mild and advanced PD showed no significant correlation with T2DM in Iraqi patients, while moderate status was more frequent in these patients than in non-diabetic patients.^43^ Many factors may play a role in the development of PD in DM patients. Age, sex, and oral health status are the most effective factors when old age, men, and poor oral health, respectively, increase PD development in DM patients.^44^ The severity of PD is greater in T2DM patients over 5 years of age compared to those under 5 years of age.^39^

From another perspective, PD is considered to be a risk factor for DM and may be associated with other DM complications.^25^ The prevalence of T2DM in periodontitis patients is greater (75.6%) than in those who do not have periodontitis (22.4%).^45^ In addition, PD has a negative impact on DM, as demonstrated by numerous studies in which the microbes responsible for PD may induce an inflammatory response that increases insulin resistance and blood sugar.^12,32^ Other mechanisms have proposed that PD can induce insulin resistance in a manner similar to that of obesity.^32^

The correlation between DM and PD may be insignificant in some cases. A comparison between Japanese patients with DM and those without DM revealed no clear association between DM and oral health status and also predicated a little effect of diabetes on the progress of PD.^46^

Gum disease, a different type of PD, is also affected by DM. Their prevalence is high in patients with DM compared to healthy people.^47,48^ Gingivitis is significantly higher among T1DM patients than in the healthy group.^41^ It is a mild form of PD that can be characterized by the presence of local erythema, swelling, and easy bleeding.^2,47^ On the other hand, gum disease can play a role in increasing blood sugar levels, which can increase the risk of developing DM or the difficulty of controlling. Treatment of gum disease is found to decrease blood sugar in patients with T2DM.^47^ The mechanism by which blood sugar is increased by gum disease may relate to inducing the immune system to produce harmful molecules by microbes that release themselves from gum tissue during chewing or brushing and these molecules can affect other sites of the human body such as pancreatic sites and cause loss of sugar control.^47^

Oral candidiasis is the third type of infection in DM patients after PD and dental caries. It develops easily in patients with poorly controlled diabetes and those with long-standing DM.^2,25^ The prevalence of oral candidiasis in DM patients is estimated to be 49%.^49,50^ Many factors can promote the development of oral candidiasis in patients with diabetes. Wearing a denture is a significant risk factor that increases the frequency of oral candidiasis in DM patients.^50^ Denture stomatitis as a common type of candidal infection associated with the wearing of denture is found in a higher rate in patients with diabetes compared to people without diabetes.^51^ Other factors are also associated with the risk of DM, such as elevated glycosylated hemoglobin above 12%, age, duration of DM, and chronic renal disease.^49,50,52,53^ On the other hand, some DM conditions may not have a role in the development of oral candidiasis in diabetic patients such as fasting glucose, urinary glucose levels, antibiotic used, white blood cell count or differential, glycosylated hemoglobin value, and the presence of diabetic retinopathy.^22,49,53-55^

Some cases of oral candidiasis have not shown any significant association with DM. Seven studies reported that patients with DM had a similar risk of developing oral candidiasis compared to those without DM.^51^ Yeasts present in the oral cavity of children with T1DM are not found to be affected by diabetes, and a high number of *C. albicans* is observed in non-diabetic children.^56^

### Impact of Diabetes Mellitus on Microbiota of Oral Cavity

The conditions created by DM have a variable effect on the diversity and distribution of various microbiota in the oral cavity.^17^ This effect of DM can change the behavior of microbial species that have colonized different sites of the oral cavity. Since this change will disrupt the correlation between the oral microbiota and its location, one or more oral diseases may occur.^2,11^ According to the animal model experiment, DM increases the pathogenicity of the oral microbiota, as indicated by the development of various oral diseases, by increasing the activities of inflammation, osteoclastogenesis, and periodontal bone loss.^16^

The predominant condition of the oral microbiota may easily change when DM is present. Such an effect of DM can lead to 3 results which are no effect, increase in the effect, and decrease in the effect in divers or the number of oral microbial flora. In general, diabetes frequently increases the number and variety of the oral microbiota. Gram-positive bacteria are the most abundant in patients with DM than Gram-negative bacteria.^38^ The variation of oral microbiota is mostly observed after comparing DM patients with healthy individuals. Many studies have indicated that the number of oral bacteria is nearly greater in DM patients, especially those with poorly controlled DM, than in healthy individuals.^15,16^ It also showed that the oral cavity of the T2DM patient contains 10 times more bacteria than that of the non-diabetic patient.^14^

The dominant and high number of some species of oral microbiota can be detected in DM patients compared with healthy individuals such as *C. albicans*, hemolytic *Streptococci*, and *Staphylococcus* spp.^17^ The phylum Firmicutes with its genera *Prevotella *and *Leptotrichia* as acidogenic bacteria and *Veillonella* as aciduric bacteria was found in greater abundance in Pakistani DM patients than in the control group.^57^ The total number of species of *Streptococcus* and *Lactobacillus* isolated from supragingival plaque was higher in Thai patients with DM than in patients without DM.^34^ Many species of bacteria can only be found in DM patients, as is the case with the *Capnocytophaga* spp. that is identified in T1DM patients and not in the control group.^15,41^ It is also proven that this bacterium, in addition to *Prophyromonas* spp. and *Pseudomonas *spp., increases in number in DM cases.^16^

Diabetes mellitus can also show a negative correlation to the oral microbiota as many studies have reported. A non-significant association was observed between species diversity and T2DM glycemic level.^58^ The anaerobic and aerobic oral microbial flora also showed no significant differences between patients with DM and the control group.^41^ Three species of oral pathogens, namely *Fusobacterium *spp., *Porphyromonas gingivalis*, and *Tannerella forsythia* may be isolated without any difference from patients with or without DM.^59^ A comparison between DM and non-DM patients revealed no differences in the presence of *Prevotella copri*, *Alloprevotella rava*, and *Ralstonia pickettii* between 2 groups.^18^ The abundance of phylum *Actinobacteria* sp. is found without significant difference between patients with DM and the healthy control group.^57^ This was also true for the subgingival microflora that showed no difference in observation between DM and non-DM.^25^

Some oral microbiota may be eliminated or reduced when DM is present. *Proteobacteria* species are not found in DM patients but are clearly seen in healthy individuals.^18,19^ The rate of *Bifidobacteria* spp. is reduced in T1DM patients compared to patients without DM.^14^

Many oral microbiota display a changing appearance in harmony with glucose levels in DM patients. Bacteria associated with dental surfaces in the form of biofilm, such as *Streptococcus mutans *and Lactobacilli, can demineralize teeth under conditions of high levels of glucose and acid causing PD.^11^ Among 44 oral microbiota taxa in DM patients, 27 taxa increased with a higher glucose change and 17 taxa with low glucose change.^60^ Some species of oral bacteria that positively associate with DM may serve as an indicator to predict DM as they increase in number. A total of 9 oral bacterial taxa can be used as a reference for predicting changes in glucose levels when glucose is higher as with *Stomatobaculum *sp. and *Atopobium *spp. or low as with *Leptotrichia *spp.^60^ The oral species that are most likely to predict the development of DM are* Megaspaera micronuciformis*, *Eubacterium sulci*, *Lactobacilus mucosae*, *Lactobacilus paraplantarum*, *Phocaecola abscessus*, *Acinetobacter nosocomialis*, *Solobaterium moorei*, *Shuttleworthia satelles*, *Streptococcus acidominimus, Enterobacter cloacae, Klebsiella oxytoca, Pseudomonas aeruginosa*, *Streptococcus salivarius*, *Actinobacillus actinomycetemcomitans*, *Bacteroides oralis, Staphylococcus aureus*, and *Fusobacteria*.^15,18,19,38^

The study of the impact of DM on the variety and number of oral microbiota is not only dependent on comparison with healthy individuals but also with patients with one or more oral diseases. A large number of various bacteria, including *Streptococcus* spp. and *Lactobacillus* spp., have been positively associated with DM.^34^ The number of *Lactobacillus* spp. in saliva and supragingival plaque was also very high in DM patients with active caries compared to those without caries.^34^ Divers bacteria of Gram-negative that are motile, capnophilic, and anaerobic and Gram-positive as* Streptococcus* spp. and Actinomyces showed an increase in number in DM patients with periodontitis compared to non-diabetic patients with periodontitis.^17^ The proportion of *Porphyromonas* spp. and *Treponema* spp. in patients with PD and T2DM was found greater than in patients with T2DM alone.^18^

The composition of the oral microbiota in DM patients may change depending on the type of dental disease.^19^ Periodontal diseases are generally caused by many of the predominant pathogens of the oral microbiota, which belong mainly to 3 aerobic species, including *A. actinomycetemcomitans*,* Campylobacter rectus*, and *Eikenella corrodens* and 7 anaerobic species, including *P. gingivalis*,* Bacteroides forsythus*,* Treponema denticola*,* Prevotella intermedia*,* Fusobacterium nucleatum*,* Eubacterium*, and *spirochetes*.^16,56^ Such a different species could be changed in the presence of DM. A comparison between a DM and a non-DM patient who both have dental caries, periodontitis, and gingivitis revealed isolation of 82 bacterial strains from DM patients compared with 60 strains from non-diabetic patients with a high ratio of isolation from the periodontitis group.^38^

Gingivitis is mostly caused by anaerobic Gram-negative bacteria that is responsible for inflammatory reaction in the gingival and bony tissues.^26^ The diversity and number of oral microbiota associated with gingivitis in patients with DM are higher than in patients with no diabetes.^15^ An abundance of *Bacteroides* was found to be more associated with bleeding gingivitis in DM patients, while *Actinobacteria* sp. was less.^19^ Ten species of oral microbial flora from gingivitis lesion in DM patients, particularly those with poorly controlled DM, were more numerous than in the control group.^15^ These species include *Streptococcus constellatus*, *S. acidominimus*, *Streptococcus oralis*, *Enterococcus faecalis*, *S. aureus*, *E. cloacae*, *K. oxytoca*, *Escherichia coli*, *P. aeruginosa*, and *C. albicans*.

Saliva is a suitable environment for a wide range of oral microbiota and may regulate them by its content of ionic, gaseous, and organic components.^10^ This microbiota of saliva has shown a significant increase in diversity and numeration in DM patients compared to people without diabetes.^18^ Many factors may have a role to play in increasing the microbial content of saliva in DM, such as xerostomia, high salivary glucose, low lysozyme, and uncontrolled DM.^41,61^ A high number of *Streptococcus mutans* and *Lactobacilli* has been observed in the saliva of patients with T2DM and the presence of a significant correlation between *Streptococcus mutans* and salivary flow and salivary buffer capacity.^61^ A greater diversity of saliva microbiota was observed in DM patients with PD than in healthy individuals.^18^

### Diabetes Mellitus and Oral ***Candida*** Species

*Candida *spp., which is one of the normal oral microbiota, can also be affected by DM conditions and transform to pathogenic organisms. The prevalence of *Candida* spp. is often higher among patients with DM than those without DM.^2,11,20-24,55^ Variation between *Candida *species in the oral cavity is seen most of the time significantly between the DM patient and the healthy group,^54^ while in some cases, there is no significant variation.^20^ The DM can encourage different species of *Candida* to become pathogenic through many associated factors such as xerostomia, low salivary flow, higher salivary glucose level, and microvascular degeneration.^2,20^ Poorly controlled DM is also a factor in improving colonization and increasing the number of *Candida* spp. in the oral cavity of DM patients.^62,63^ Immunosuppressed conditions may be added to be the most important factor inducing the oral pathogenicity of *Candida* spp. in DM patients through the failure of the immune system to control excessive growth of this fungus that is enhanced by DM predisposing factors.^2,11,12^ Other factors that are not related to DM may also allow oral candidiasis infection in patients with DM such as smoking, carrying dentures, female, and prolonged use of antibiotics or indwelling medical devices.^2,12,23^ Thus, treating oral candidiasis in patients with DM is more complicated than in healthy people.^2^

*C. albicans *is the most common species of *Candida* in diabetic patients compared to non-diabetic individuals.^21-24^ Other species of *Candida *have also been diagnosed as common yeast in the oral cavity of the patient with DM such as *Candida krusei*,* Candida glabrata*,* Candida tropicalis*, and* Candida parapsilosis*.^21,23^ *Candida dubliniensis, *a species close to* C. albicans *with difficult differentiation, is also present with a high prevalence in patients with DM.^22^ Colonization of *C. albicans* in the oral cavity is diagnosed in 56% of T2DM patients compared to 30 in the non-DM group.^53^ Duration of diabetes is an effective addition factor that increases colonization of *C. albicans* in the oral cavity.^55^ Resistance to antifungal agents may also increase in *C. albicans* because of DM conditions.^62^

## Conclusions

The oral microbiota community may have a different response to the presence of DM. Most of this response can lead to unwanted outcomes represented by various oral diseases such as dental caries, PD, and oral candidiasis. Some oral microbiota species may increase in number or diversity as a result of DM, while others may be decreased or unaffected. Increased microbial growth is commonly seen in DM due to elevated blood glucose levels which disrupt the balance among different species in the oral community. Thus, many diseases can develop in various parts of the oral cavity resulting from the inducing role of DM in increasing the pathogenesis of some oral microbiota. Fungal species of the oral microbiota are affected by DM in the same way as bacteria. Oral candidiasis which is mainly caused by *C. albicans* is the most frequent type of fungal infection of the oral cavity due to DM.

## References

[1] Kosti M Kanakari M . Education and diabetes mellitus. Health Sci J. 2012;6:654 662.

[2] Rodrigues CF Rodrigues ME Henriques M . *Candida* sp. Infections in patients with diabetes mellitus. J Clin Med. 2019;8(1):76. 10.3390/jcm8010076) PMC6352194 30634716

[3] Riaz S Diabetes mellitus. Sci Res Essays. 2009;4:367 373.

[4] World Health Organization. Classification of diabetes mellitus. Department of management of Noncommunicable Diseases, Disability, Violence and Injury Prevention. Available at: https://www.who.int/publications/i/item/classification-of-diabetes-mellitus; 2019.

[5] World Health Organization. Definition, diagnosis and classification of diabetes mellitus and its complications. Part 1: diagnosis and classification of diabetes mellitus. Department of Noncommunicable Disease Surveillance. Available at: https://apps.who.int/iris/handle/10665/66040; 1999.

[6] Kilian M Chapple ILC Hannig M et al. The oral microbiome-an update for oral healthcare professionals. Br Dent J. 2016;221(10):657 666. 10.1038/sj.bdj.2016.865) 27857087

[7] Majumdar S Singh AB . Normal microbial flora of oral cavity. Adv Med Dent Sci Research. 2014;2:62 66.

[8] Xiao J Fiscella KA Gill SR . Oral microbiome: possible harbinger for children's health. Int J Oral Sci. 2020;12(1):12. 10.1038/s41368-020-0082-x) PMC7190716 32350240

[9] Mrinalini MNH Dysbiosis of oral microflora: a review. Nitte Univ J Health Sci. 2018;8:34 39.

[10] Schuster GS Oral flora and pathogenic organisms. Infect Dis Clin North Am. 1999;13(4):757 774. 10.1016/s0891-5520(05)70107-0) 10579107

[11] Verhulst MJL Loos BG Gerdes VEA Teeuw WJ . Evaluating all potential oral complications of ­diabetes mellitus. Front Endocrinol. 2019;10:56. 10.3389/fendo.2019.00056) PMC6439528 30962800

[12] Rohani B Oral manifestations in patients with diabetes mellitus. World J Diabetes. 2019;10(9):485 489. 10.4239/wjd.v10.i9.485) 31558983 PMC6748880

[13] Reddy SS Kripal K Nair SK Kumar PA . Prevalence and incidence of periodontal diseases in diabetes mellitus patients. EC Dent Sci. 2017;9:173 181.

[14] Shillitoe E Weinstock R Kim T et al. The oral microflora in obesity and type-2 diabetes. J Oral Microbiol. 2012;4:19013. 10.3402/jom.v4i0.19013) PMC3485401 23119124

[15] Hsaine S Fethi FZ Charof R Ounine K . Microbiological study of oral flora in diabetic patients with gingivitis. Int J Pharm Pharm Sci. 2018;10(6):113 116. 10.22159/ijpps.2018v10i6.26295)

[16] Graves DT Corrêa JD Silva TA . The oral microbiota in modified by systemic diseases. J Dent Res. 2019;98(2):148 156. 10.1177/0022034518805739) 30359170 PMC6761737

[17] Kulshrestha R Neral A Srinivasa TS Baig SA . Comparison of oral microflora of diabetic and non-diabetic patients with periodontitis. J Pure Appl Microbiol. 2011;5:883 886.

[18] Sun X Li M Xia L et al. Alteration of salivary microbiome in periodontitis with or without type-2 diabetes mellitus and metformin treatment. Sci Rep. 2020;10(1):15363. 10.1038/s41598-020-72035-1) PMC7506544 32958790

[19] Matsha TE Prince Y Davids S et al. Oral microbiome signatures in diabetes mellitus and periodontal disease. J Dent Res. 2020;99(6):658 665. 10.1177/0022034520913818) 32298191

[20] de la Rosa-García E Miramontes-Zapata M Sánchez-Vargas LO Mondragón-Padilla A . Oral colonisation and infection by *Candida* sp. in diabetic and non-diabetic patients with chronic kidney diseases on dialysis. Nefrologia. 2013;33(6):764 770. 10.3265/Nefrologia.pre2013.Aug.11790) 24241363

[21] Mohammadi F Javaheri MR Nekoeian S Dehghan P . Identification of *Candida* species in the oral cavity of diabetic patients. Curr Med Mycol. 2016;2(2):1 7. 10.18869/acadpub.cmm.2.2.4) PMC5490298 28681013

[22] Zomorodian K Kavoosi F Pishdad GR et al. Prevalence of oral *Candida* colonization in patients with diabetic mellitus. J Mycol Med. 2016;26(2):103 110. 10.1016/j.mycmed.2015.12.008) 26879707

[23] Sampath A Weerasekera M Dilhari A et al. Type 2 diabetes mellitus and oral *Candida* colonization: analysis of risk factors in a Sri Lankan cohort. ACTA Odontol Scand. 2019;77(7):508 516. 10.1080/00016357.2019.1607547) 31145647

[24] Mamari A Hegami M Sophiany N et al. Prevalence of oral candidiasis among diabetics-non diabetics patients and evaluate the contribution of risk factors in IBB city. Int J Adv Res. 2017;5(12):1372 1380. 10.21474/IJAR01/6096)

[25] Lamster IB Lalla E Borgnakke WS Taylor GW . The relationship between oral health and diabetes mellitus. J Am Dent Assoc. 2008;139(suppl):19S 24S. 10.14219/jada.archive.2008.0363) 18809650

[26] Negrato CA Tarzia O Jovanovič L Chinellato LE . Periodontal diseases and diabetes mellitus. J Appl Oral Sci. 2013;21(1):1 12. 10.1590/1678-7757201302106) 23559105 PMC3881811

[27] Khatib OMN Guidelines for the Prevention, Management and Care of Diabetes Mellitus. WHO. EMRO Technical Publications Series 32; 2006. Available at: https://apps.who.int/iris/handle/10665/119799

[28] Kane SF The effects of oral health on systemic health. Gen Dent. 2017;65(6):30 34. 29099363

[29] American Diabetes Association. Diagnosis and classification of diabetes mellitus. Diabetes Care. 2014;37(suppl 1):S81 S90. 10.2337/dc14-S081) 24357215

[30] Baynest HW Classification, pathophysiology, diagnosis and management of diabetes mellitus. Baynes J Diabetes Metab. 2015;6:5. 10.4172/2155-6156.1000541)

[31] López ME Colloca ME Páez RG Schallmach JN Koss MA Chervonagura A . Salivary characteristics of diabetic children. Braz Dent J. 2003;14(1):26 31. 10.1590/s0103-64402003000100005) 12656461

[32] Kumar M Mishra L Mohanty R Nayak R . Diabetes and gum diseases: the diabolic duo. Diabetes Metab Syndr Clin Res Rev. 2014;8:255 258. 10.1016/j.dsx.2014.09.022 25450824

[33] Sarmamy HM Saber SM Majeed VO . The influence of type 1 diabetes mellitus on dentition and oral health of children and adolescents attending two diabetic centers in Erbil city. Zanco J Med Sci. 2012;16(3):204 212. 10.15218/zjms.2012.0036)

[34] Kampoo K Teanpaisan R Ledder RG McBain AJ . Oral bacterial communities in individuals with type 2 diabetes who live in Southern Thailand. Appl Environ Microbiol. 2014;80(2):662 671. 10.1128/AEM.02821-13) 24242241 PMC3911101

[35] Wu CZ Yuan YH Liu HH et al. Epidemiologic relationship between periodontitis and type 2 diabetes mellitus. BMC Oral Health. 2020;20(1):204. 10.1186/s12903-020-01180-w) PMC7353775 32652980

[36] Thaper S Thaper T Priya VV Thaper R Thaper R . Prevalence of periodontitis in diabetic and non-diabetic patients. Asian J Pharm Clin Res. 2016;9:329 331.

[37] Das M Upadhyaya V Ramachandra SS ­Jithendra KD . Periodontal treatment needs in diabetic and non-diabetic individuals: a case-control study. Indian J Dent Res. 2011;22(2):291 294. 10.4103/0970-9290.84307) 21891902

[38] Sharma M Tiwari SC Singh K Kishor K . Occurrence of bacterial flora in oral infections of diabetic and non-diabetic patients. Life Sci Res. 2011:LSMR-32. Available at: http://astonjournals.com/lsmr.

[39] Khader YS Albashaireh ZS Hammad MM . Periodontal status of type 2 diabetics compared with nondiabetics in North Jordan. East Mediterr Health J. 2008;14(3):654 661. 18720630

[40] Apoorva SM Sridhar N Suchetha A . Prevalence and severity of periodontal disease in type 2 diabetes mellitus (non-insulin-dependent diabetes mellitus) patients in Bangalore city: an epidemiological study. J Indian Soc Periodontol. 2013;17(1):25 29. 10.4103/0972-124X.107470) 23633768 PMC3636938

[41] Pinducciu G Micheletti L Piras V et al. Periodontal disease, oral microbial flora and salivary antibacterial factors in diabetes mellitus type 1 patients. Eur J Epidemiol. 1996;12(6):631 636. 10.1007/BF00499463) 8982624

[42] Abbas DK Periodontal treatment needs of type 2 diabetic Iraqi patients. *MDJ* . 2011;8:79 85.

[43] Mansour AA Abd-Al-Sada N . Periodontal disease among diabetics in Iraq. MedGenMed. 2005;7(3):2. Available at: https://www.ncbi.nlm.nih.gov/pmc/articles/PMC1681616/ PMC1681616 16369228

[44] Han K Park JB . Clinical implications of age and sex in the prevalence of periodontitis in Korean adults with diabetes. Exp Ther Med. 2018;15(4):3865 3873. 10.3892/etm.2018.5880) 29556264 PMC5844085

[45] Awuti G Younusi K Li L Upur H Ren J . Epidemiological survey on the prevalence of periodontitis and diabetes mellitus in Uyghur adults from rural Hotan area in Xinjiang. Exp Diabetes Res. ID. 2012;2012:758921. 10.1155/2012/758921) PMC3150776 21826136

[46] Ueno M Takeuchi S Oshiro A Shinada K Ohara S Kawaguchi Y . Association between diabetes mellitus and oral health status in Japanese adults. Int J Oral Sci. 2010;2(2):82 89. 10.4248/IJOS10025) 20737934 PMC3733588

[47] American Dental association. Gum disease can raise your blood sugar level. JADA. 2013;144:860. Available at: http://jada.ada.org. Accessed July 2013. 10.14219/jada.archive.2013.0199 23813269

[48] Latti BR Kalburge JV Birajdar SB Latti RG . Evaluation of relationship between dental caries, diabetes mellitus and oral microbiota in diabetics. J Oral Maxillofac Pathol. 2018;22(2):282. 10.4103/jomfp.JOMFP_163_16) PMC6097371 30158791

[49] Hill LV Tan MH Pereira LH Embil JA . Association or oral candidiasis with diabetic control. J Clin Pathol. 1989;42(5):502 505. 10.1136/jcp.42.5.502) 2732344 PMC1141957

[50] Jabber WF Adi QA . Diabetic control and the occurrence of oral candidiasis. Ai-Anbar Med J. 2011;9:43 49.

[51] Martorano-Fernandes L Dornelas-Figueira LM Marcello-Machado RM et al. Oral candidiasis and denture stomatitis in diabetic patients: systematic review and meta-analysis. Braz Oral Res. 2020;34:e113. 10.1590/1807-3107bor-2020.vol34.0113) 32965459

[52] Nguyen TD Nguyen TT Tran QV . The incidence of oral candidiasis in patients with diabetes mellitus: a cross-sectional study in Southern Vietnam. J Crit Rev. 2020;7:82 86.

[53] Amin AM Sadiq N Saeed CH . Isolation of *Candida albicans* from oral cavity of type II diabetic subjects and its relationship to total and differential white blood cell count. Zanco J Med Sci. 2014;18(3):833 838. 10.15218/zjms.2014.0042)

[54] Bartholomew GA Rodu B Bell DS . Oral candidiasis in patients with diabetes mellitus: a thorough analysis. Diabetes Care. 1987;10(5):607 612. 10.2337/diacare.10.5.607) 3677980

[55] Jafari AA Khanpayah E Ahadian H . Comparison the oral *Candida* carriage in type 2 diabetic and non diabetics. Jundishapur J Microbiol. 2013;6(7):e8495. 10.5812/jjm.8495)

[56] Costa AL Silva BMA Soares R et al. Type 1 diabetes in children is not a predisposing factor for oral yeast colonization. Med Mycol. 2017;55(4):358 367. 10.1093/mmy/myw092) 27664993

[57] Kori JA Saleem F Ullah S Azim MK . Characterization of oral bacteriome dysbiosis in type 2 diabetic patients. MedRxiv. 2020:1 19. 10.1101/2020.04.09.20052613)

[58] Tam J Hoffmann T Fischer S Bornstein S Gräßler J Noack B . Obesity alters composition and diversity of the oral microbiota in patients with type 2 diabetes mellitus independently of glycemic control. PLOS ONE. 2018;13(10):e0204724. 10.1371/journal.pone.0204724) PMC6166950 30273364

[59] Al-Rawi N Al-Marzooq F . The relation between periodontopathogenic bacterial levels and resistin in the saliva of obese type 2 diabetic patients. J Diabetes Res. 2017. 10.1155/2017/2643079) PMC5613684 29138754

[60] Demmer RT Trinh P Rosenbaum M et al. Subgingival microbiota and longitudinal glucose change: the oral infections, glucose intolerance and insulin resistance study (ORIGINS). J Dent Res. 2019;98(13):1488 1496. 10.1177/0022034519881978) 31623509 PMC6873283

[61] Almusawi MA Gosadi I Abidia R et al. Association between salivary factors and cariogenic bacteria in type-2 diabetes patients. J King Saud Univ Sci. 2020;32(5):2617 2621. 10.1016/j.jksus.2020.05.002)

[62] Bhuyan L Hassan S Dash KC Panda A Behura SS Ramachandra S . *Candida* species diversity in oral cavity of type 2 diabetic patients and their *in vitro* antifungal susceptibility. Contemp Clin Dent. 2018;9(Suppl 1):S83 S88. 10.4103/ccd.ccd_70_18) 29962770 PMC6006883

[63] Fenn SM Narayanan M Jacob M . Prevalence of oral *Candida* in saliva of uncontrolled and controlled type 2 diabetes mellitus patients-beyond reasonable doubt? SRM J Res Dent Sci. 2019;10(1):1 6. 10.4103/srmjrds.srmjrds_48_18)

